# Hematein, a casein kinase II inhibitor, inhibits lung cancer tumor growth in a murine xenograft model

**DOI:** 10.3892/ijo.2013.2087

**Published:** 2013-09-04

**Authors:** MING-SZU HUNG, ZHIDONG XU, YU CHEN, EMMANUEL SMITH, JIAN-HUA MAO, DAVID HSIEH, YU-CHING LIN, CHENG-TA YANG, DAVID M. JABLONS, LIANG YOU

**Affiliations:** 1 Thoracic Oncology Laboratory, Department of Surgery, Comprehensive Cancer Center, University of California, San Francisco, CA 94115, USA;; 2 Division of Pulmonary and Critical Care Medicine, Chang Gung Memorial Hospital, Chiayi branch;; 3 Department of Medicine, College of Medicine, Chang Gung University, Taoyuan;; 4 Department of Respiratory Care, Chang Gung University of Science and Technology, Chiayi Campus, Chiayi, Taiwan, R.O.C.;; 5 Department of Molecular Medicine, College of Medicine, University of South Florida, Tampa, FL;; 6 Life Sciences Division, Lawrence Berkeley National Laboratory, University of California, Berkeley, CA, USA;; 7 Division of Pulmonary and Critical Care Medicine, Chang Gung Memorial Hospital, Taoyuan branch;; 8 Department of Respiratory Care, College of Medicine, Chang Gung University, Taoyuan, Taiwan, R.O.C.

**Keywords:** hematein, casein kinase II, Wnt, lung cancer, xenograft

## Abstract

Casein kinase II (CK2) inhibitors suppress cancer cell growth. In this study, we examined the inhibitory effects of a novel CK2 inhibitor, hematein, on tumor growth in a murine xenograft model. We found that in lung cancer cells, hematein inhibited cancer cell growth, Akt/PKB Ser129 phosphorylation, the Wnt/TCF pathway and increased apoptosis. In a murine xenograft model of lung cancer, hematein inhibited tumor growth without significant toxicity to the mice tested. Molecular docking showed that hematein binds to CK2α in durable binding sites. Collectively, our results suggest that hematein is an allosteric inhibitor of protein kinase CK2 and has antitumor activity to lung cancer.

## Introduction

Casein kinase II (CK2), which is pleiotropic aserine/threonine protein kinase composed of 2 catalytic subunits (αα, α′α′ or αα′) and 2 regulatory subunits (β), is ubiquitously expressed and highly conserved in cells. Through phosphorylation to more than 300 proteins in cells, CK2 is an important regulator of intracellular signalling pathways (1), and exerts many roles in cellular processes, including gene expression, protein synthesis, cell proliferation and apoptosis (2).

CK2 has been regarded as a potential candidate for targeted therapy for cancers because dysregulation of CK2 in association with other proteins increases oncogenic potential of cells (3). In transgenic mice, overexpression of CK2α subunits is reportedly associated with the development of lymphoma (4) and adenocarcinomas of the mammary gland (5). Overexpression of CK2 has been reported in a variety of human cancers, including acute myeloid leukaemia (6), mammary gland (5), prostate (7), lung (8), head and neck (9), and kidney cancer (10), and also correlates with metastatic potential, undifferentiated histological type and poor clinical outcome in human cancers. Various CK2 inhibitors have been discovered. For example, TBB (4,5,6,7 tetrabrome benzotriazole) (11) and its derivatives (12,13) have been shown to induce apoptosis in human cancer cells. A potent and selective orally bioavailable small molecule inhibitor of CK2, CX-4945, is being tested in a clinical trial (14).

We previously showed that a novel CK2 inhibitor, hematein (3,4,10,6a-tetrahydroxy-7, 6 adihydroindeno [2,1-c] chroman-9-one), inhibited cancer cell growth and was noted to have a high selectivity towards CK2 among a kinase panel of 48 kinases (15). Hematein is a natural compound from *Caesalpinia sappan* with a molecular weight of 300.26 Da, and has been used in oriental medicine as an analgesic and anti-inflammatory agent (16). It is also used in histochemical staining (17). Hematein has the *in vitro* IC_50_ value of 0.74 *μ*M on CK2 kinase activity, which is comparable to other CK2 inhibitors (12). However, the effect of hematein on tumor growth in animal models and the binding mode of hematein to CK2 remain unknown. We therefore examined the inhibitory effects of hematein on lung cancer tumor growth in a murine xenograft model and used molecular docking to elucidate how hematein binds to CK2.

## Materials and methods

### Cell culture

A427 (HTB-53) cell line was purchased from American Type Culture Collection (Manassas, VA). Cells were grown in complete growth medium (Roswell Park Memorial Institute) supplemented with 10% fetal bovine serum, 10 units/ ml penicillin and 10 *μ*g/ml streptomycin at 37°C and 5% CO_2_.

### Cell viability assay

The toxicity of hematein was evaluated by CellTiter-Glo luminescent cell viability assay (Promega, Madison, WI) was used to evaluate the cytotoxicity of hematein according to the manufacturer’s manual (15). In brief, after incubation with indicated amount of compounds for 48 h, 100 *μ*l of the CellTiter-Glo reagent was added directly to culture wells. The luminescence produced by the luciferase-catalyzed reaction of luciferin and ATP was measured using a luminometer.

### Colony formation assay

A427 lung cancer cells (5×10^2^) were plated in 10 cm culture dishes and incubated in complete medium with indicated concentrations of hematein (Sciencelab. com, Inc., Houston, TX) for 14 days. The colonies were then stained with 0.1% crystal violet, and colonies of greater than 50 cells were counted. Results were expressed as relative colony formation: percentage of the number of colonies relative to the control group. Three independent experiments were performed.

### Western blot analysis

After treatment with indicated concentrations of hematein for 48 h, whole cell proteins were extracted from A427 cells with M-PER Mammalian Protein Extraction Reagent (Pierce, Rockfold, IL) added to Phosphatase Inhibitor Cocktail Set II (Calbiochem, San Diego, CA) and Complete Protease Inhibitor Cocktails (Roche, Switzerland) according to manufacturer’s protocols. Proteins were separated on 4–15% gradient sodium dodecyl sulfate (SDS)-polyacrylamide gels and transferred to Immobilon-P membranes (Millipore, Billerica, MA). The following primary antibodies were used: Akt, PARP, survivin (Cell Signaling Technology, Danvers, MA), phospho-Akt S129 (Abcam Inc., Cambridge, MA) and β-actin (Sigma, St. Louis, MO). After primary antibody and antigen complexes were bound to specific secondary antibodies, an enhanced chemiluminescence (ECL) blotting analysis system (GE Healthcare Life Sciences, Piscataway, NJ) was used for antigen-antibody detection. Densitometry of western blot analysis was calculated by using ImageJ (v1.44m for Windows, National Institutes of Health).

### Transient transfection and luciferase reporter assay

The TOP/ FOP Flash reporter assay was performed to evaluate the TCF/LEF transcriptional activity induced by the Wnt canonical pathway. Three independent transfection experiments were performed in triplet using the Lipofectamine 2000 (Invitrogen, Carlsbad, CA, USA) according to the manufacturer’s instructions. The A427 cells were transfected with 8 *μ*g Super 8×TOPflash or 8 *μ*g Super 8×FOPflash plasmid (a kindly gift from Professor Randall Moon, Howard Hughes Medical Institute and Department of Pharmacology, University of Washington, Seattle, WA, USA), the pRL-TK plasmid (Promega) was co-transfected to normalize for transfection efficiency. Twenty-four hours after transfection, cells were treated with hematein (50 or 100 *μ*M) for 24 h. Luciferase activity was then assayed using the Dual-Luciferase^®^ Reporter Assay System (Promega) with a luminometer.

### Murine xenograft model

After approval was obtained from our institutional animal care and use committee, groups of 6 female athymic BALB/c nude mice (6-week-old), received subcutaneous injections of 4×10^6^ A427 cells in the flank area with a volume of 100 *μ*l PBS with 25% matrigel (BD Biosciences, Bedford, MA). Seven days later, tumors had formed. The mice then received intraperitoneal injections twice a week with 50 mg/kg of hematein or 5% DMSO dissolved in PBS as the control. Tumor size was determined twice a week for 6 weeks, and tumor volume was calculated on the basis of width (*x*) and length (*y*): *x*^2^*y*/2, where *x* < *y*. Seven weeks after injection of A427 lung cancer cells, mice were sacrificed. The heart, liver, lung and kidney were resected, fixed and stained with hematoxylin and eosin according to standard methods. All slides were reviewed by a pathologist and were were photographed using a Zeiss AxioCam camera with Zeiss AxioVision software.

### Immunohistochemistry

The formalin-fixed and paraffin-embedded tumors were sliced into 5 *μ*m sections and were deparaffinized in xylene and then rehydrated in graded alcohol. Antigen retrieval was performed by steaming the tissue sections in citrate buffer (10 mM, 0.05% Tween-20, pH 6.0) for 20 min. Slides were then washed in TBS plus 0.025% Triton X-100, blocked in 10% normal serum with 1% BSA in TBS for 2 h at room temperature, and then incubated in the primary antibody overnight at 4°C. The rabbit polyclonal cleaved caspase-3 antibody (Cell Signaling, Boston, MA) was used as primary antibody at a 1–300 dilution in TBS with 1% BSA. Following TBST washes, endogenous peroxidase activity was then quenched with 0.3% hydrogen peroxide in TBS. Mouse and Rabbit Specific HRP/DAB (ABC) detection IHC kit (Abcam) kit was then used according to the manufacturer’s protocol. Detection was achieved using a biotinylated anti-rabbit secondary antibody and DAB chromogen. The sections were counterstained with hematoxylin before being mounted with organic media and glass slides.

### Molecular docking of hematein to CK2α

DOCK 3.5.54 was used to predict the binding pose of hematein in both the canonical ATP binding site and the allosteric DRB site of CK2α (18–20). DRB (5,6-dichloro-1-b-D-ribofuranosylbenzimidazole) was used to generate the docking environment and matching spheres. The most favourable conformation was chosen from four predicted conformations of hematein against each site. The docking results were further verified by another docking program, Accelrys Discovery Studio 2.5.

### Statistical analysis

The data shown represent mean values ± standard error of mean (SEM). Student’s t-test was used to compare tumor size. Statistical analysis was carried out using SPSS (version 14.0, Chicago, IL). Two-sided p-values <0.05 were considered statistically significant.

## Results

### Hematein inhibits cells growth, and CK2-specific Akt phosphorylation in A427 lung cancer cells

**Figure 1.**
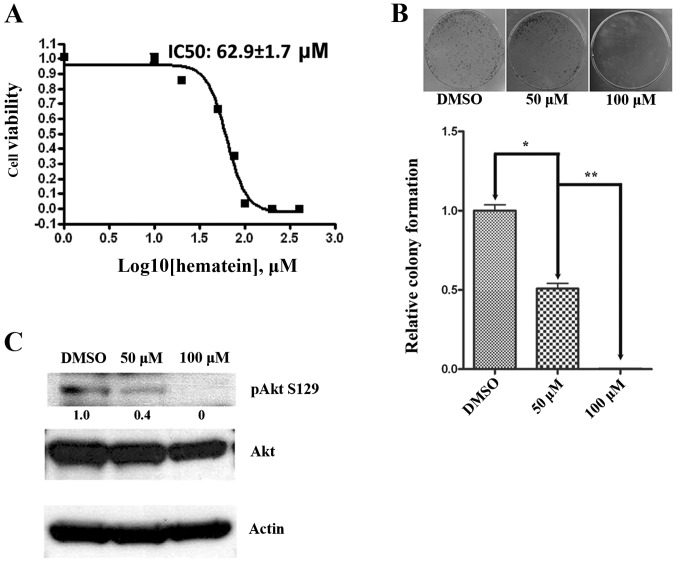
Hematein inhibits cells growth, and inhibits Akt phosphorylation in A427 lung cancer cells. (A), A427 lung cancer cells were cultured in the absence and in increasing concentrations of hematein (10–100 *μ*M) as indicated. Cellular viability (normalized to DMSO control) was measured after 48 h using CellTiter-Glo^®^ Luminescent cell viability assay. Data points represent the average of IC50 value of hematein in triplet experiments and bars indicate SD. (B), After incubation with indicated concentrations of hematein for 2 weeks, colonies of A427 lung cancer cells were stained with 0.1% crystal violet, and colonies greater than 50 cells were counted. Results are expressed as relative colony formation: percentage of the number of colonies relative to the control group. Data represent the average of three independent experiments and bars indicate SEM. ^*^p=0.0006, ^**^p=0.0001. (C), Phosphorylated Akt (Ser 129) was measured by western blot analysis. β-actin was used as an internal loading control. Band quantification was obtained by ImageJ software. Values are reported below each band and normalized to DMSO control.

The A427 lung cancer cell line was chosen for *in vitro* study because it showed the lowest IC_50_ for hematein of several cell lines that we previously tested. The IC_50_ of hematein is 62.9±1.7 *μ*M for the A427 lung cancer cell line (15) (Fig. 1A). To evaluate the inhibitory effect of hematein on cell growth, we used the anchorage-dependent colony formation assay. After culture in 50 and 100 *μ*M of hematein for 14 days, colony formation decreased significantly in A427 lung cancer cells when compared to cells treated with DMSO (Fig. 1B). Since CK2 was reported to constitutively phosphorylate and upregulate Akt S129, which is a specific phosphorylation site for CK2, *in vitro* and *in vivo* (4). The phosphorylation of Akt-S129 (Fig. 1C) was evaluated, and a dose-dependent decrease of the phosphorylation of Akt-S129 after hematein treatment was observed in A427 lung cancer cells.

### Hematein inhibits the Wnt canonical pathway, and induces apoptosis in A427 lung cancer cells

**Figure 2.**
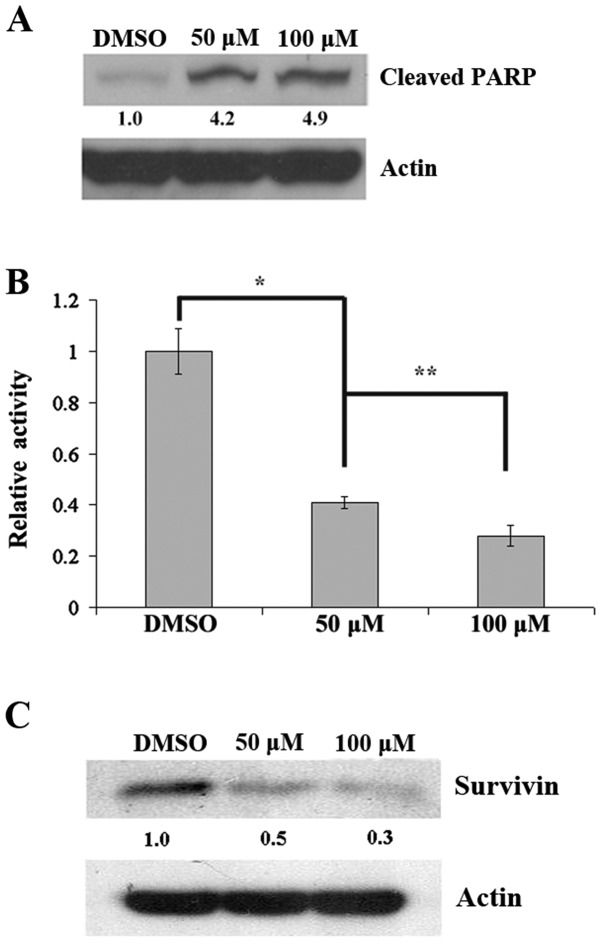
Hematein induces apoptosis and inhibits the Wnt/TCF pathway in A427 lung cancer cells. (A), After incubation with indicated concentrations of hematein for 48 h, total cell proteins were extracted from A427 lung cancer cells. Protein (50 *μ*g) was used for western blot analysis to detect the cleaved PARP. (B), The transcriptional activity of Wnt/TCF pathway in A427 cells was detected by TOP/FOP reporter assay. Results are expressed as relative activity: percentage of the activity relative to the control group. Data represent the average of three independent experiments and bars indicate SEM. ^*^p<0.0001, ^**^p=0.002. (C), Survivin was measured by western blot analysis. β-actin was used as an internal loading control. Band quantification was obtained by ImageJ software. Values are reported below each band and normalized to DMSO control.

To determine cleaved PARP as a late event in apoptosis after inhibition of CK2 by hematein, cells were treated with hematein for 48 h. We found that cleaved PARP increased in A427 lung cancer cells after treatment with hematein (Fig. 2A), which indicated increased apoptosis. In addition, down-regulation of the Wnt canonical pathway was further confirmed by a dose-dependent decrease of TOP/FOP luciferase activity (Fig. 2B) and survivin (Fig. 2C).

### Hematein inhibits tumor growth in A427 lung cancer cell xenografts

**Figure 3.**
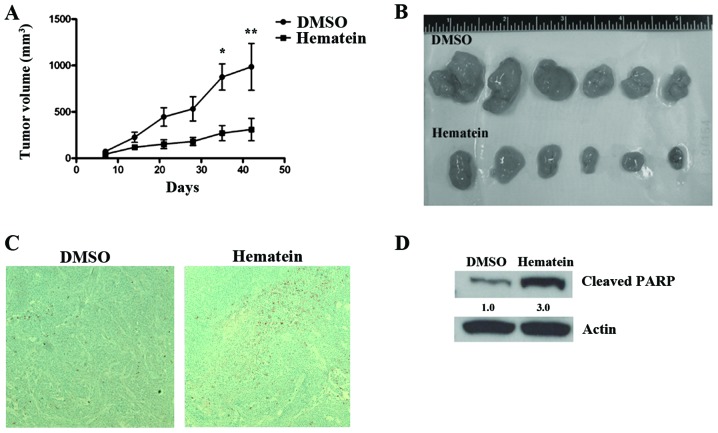
Hematein inhibits tumor growth in xenografts of A427 lung cancer cells. Groups of six, 6-week-old female BALB/c nude mice received subcutaneous injections of 4×10^5^ cells in the dorsal area in a volume of 100 *μ*l. (A), Tumor volume after treatment. DMSO or 50 mg/kg hematein was injected intraperitoneally twice a week 7 days after injection of A427 lung cancer cells. Tumor volumes were determined weekly for 6 weeks, and were calculated on the basis of tumor width (*x*) and length (*y*): *x*^2^*y*/2, where *x* < *y*. Tumor volume (mm^3^) at various times after treatment is shown. Data represent the average of tumor volume and bars indicate SEM. ^*^p=0.041, ^**^p=0.0359. (B), The sizes of A427 tumors. After the mice were sacrificed on day 42, tumors were resected. (C), Cleaved caspase-3 in A427 tumors was determined by immunohistochemical staining. (D), Total protein was extracted from tumor tissues for western blot analysis. Protein (50 *μ*g) was used for Western blot analysis to detect the cleaved PARP. β-actin was used as an internal loading control. Band quantification was obtained by ImageJ software. Values are reported below each band and normalized to DMSO control.

Since hematein inhibited growth in A427 lung cancer cells, we conducted an *in vivo* study using a murine xenograft model to evaluate the inhibitory effect of hematein on tumor growth. One week after 4×10^6^ A427 lung cancer cells were injected subcutaneously into flank areas of nude mice, hematein was injected intraperitoneally at a dosage of 50 mg/kg twice a week. Six and seven weeks after injection of A427 lung cancer cells, tumor volumes decreased significantly in the group treated with hematein when compared to the group treated with DMSO (Fig. 3A and B). Cleaved caspase-3 and cleaved PARP proteins increased in tumors treated with hematein (Fig. 3C and D).

### Hematein has minor toxicity to organs

**Figure 4.**
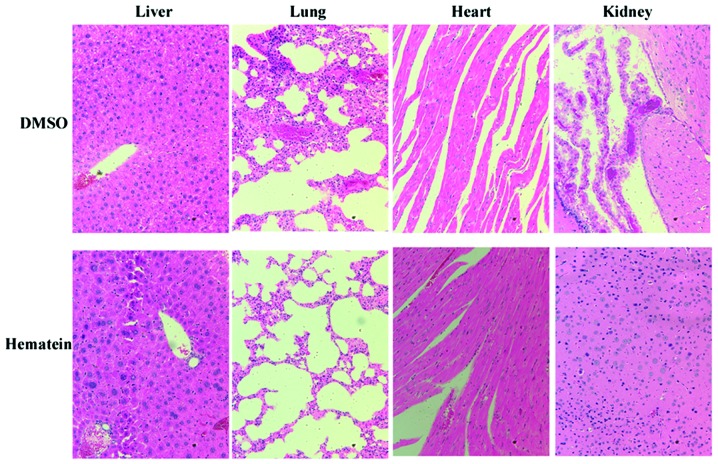
Internal organs of mice treated with DMSO or hematein in the murine xenograft model. After the mice were sacrificed on day 42, the liver, lung, heart and kidney were resected, fixed and embedded in paraffin. Samples were sliced to 5 *μ*m in thickness and stained with hematoxylin and eosin. Original magnification, ×200.

Histpathologic review of organs resected seven weeks after mice received injections of A427 lung cancer cells showed no obvious damage in heart, liver, lung and kidney (Fig. 4). No organ damage was observed in hematein treated groups when compared with DMSO treatment groups. These results showed the safety of hematein in animals studied.

### Hematein has durable binding sites to CK2

**Figure 5.**
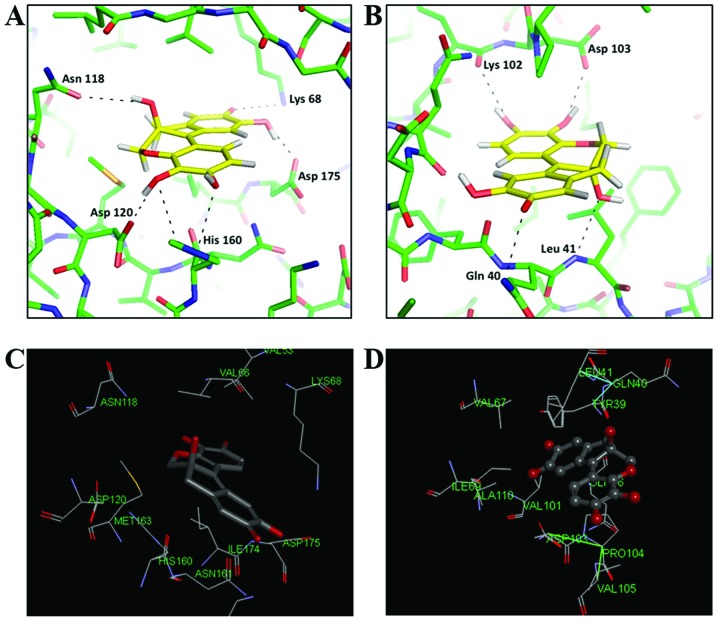
Molecular docking of hematein to CK2α. Molecular docking of hematein bound to the active site of the CK2 catalytic subunit. Tow docking programs [DOCK 3.5.54 for (A and B); Accelrys Discovery Studio 2.5 for (C and D)] were used for virtual docking. (A and C), The binding mode of hematein to the ATP binding cleft of CK2α was analyzed, in which the interactions with the most crucial amino acids are highlighted. (B and D), Hematein also docks well to an allosteric site as DRB, a well-known CK2 inhibitor. The interactions with the most crucial amino acids are highlighted.

To elucidate the binding of hematein to CK2α enzyme, virtual molecular docking was performed. Two docking programs (DOCK 3.5.54 and Accelrys Discovery Studio 2.5) were used to predict the potential docking sites of hematein to CK2α enzyme. Similar docking sites were noted by the two docking programs. Docking sites similar to those of an often-used CK2 inhibitor, 5,6-dichloro-1-b-D-ribofuranosylbenzimidazole (DRB), were noted in hematein (21). Hematein docked to the canonical ATP binding site of CK2α (Fig. 5A and C). However, hematein also docked well to an allosteric site (Fig. 5B and D), which reportedly serves as a CK2α and CK2β interface. We previously found that hematein is an ATP non-competitive inhibitor of CK2 (15), which may be explained by molecular docking of hematein to the allosteric site of CK2α preferentially in the hematein and CK2 complex.

## Discussion

Our study shows that hematein inhibited growth and Akt/ PKB Ser129 phosphorylation and increased apoptosis in lung cancer cells. Hematein also inhibited tumor growth in a murine xenograft model of lung cancer without obvious toxicity to the mice tested. Molecular docking showed durable binding sites of hematein to CK2α.

Previously, Akt/PKB Ser129 was reported to play a role in constitutive activation of Akt/PKB pathway by CK2 (22), which promotes cell survival through activation of anti-apoptotic pathways such as the NF-κB pathway and suppression of caspase activity (23). Treatment of a variety of cancer cells with cell-permeable CK2 inhibitors such as TBB, IQA and DMAT reportedly induce apotosis (11,13,24). We previously found that hematein has high selectivity for inhibition of CK2 kinase activity among a panel of protein kinases (15). Like other reported CK2 inhibitors, hematein induces apoptosis in cancer cells at least partially through inhibition of Akt/PKB pathway by down-regulation of CK2 kinase and then decreased phosphorylation of Akt/PKB Ser129. CK2 has been reported to promote cancer cell survival by increasing β-catenin-Tcf/Lef-mediated transcription and then increased expression of survivin (25). It has been reported recently that CK2α-specific enhancement of β-catenin transcriptional activity as well as cell survival may depend on Akt/PKB Ser129 hyperactivation by CK2 (26). Our study showed that in addition to inhibiting phosphorylation of Akt/PKB Ser129, hematein also inhibited the Wnt canonical pathway, which is confirmed by decreased TOP/FOP luciferase activity and survivin after treatment with hematein.

We previously reported that hematein is an ATP non-competitive and partially reversible CK2 inhibitor (15). The molecular docking analysis performed in the present study further elucidates this characteristic of hematein by showing that hematein binds to the canonical ATP binding site of CK2α, and to an allosteric site of CK2α, which is similar to the reported binding site of DRB. The allosteric site for hematein is a hydrophobic pocket at the outer surface of the N-terminal β sheet of CK2α and serves as a CK2α and CK2β interface (19). A recently reported class of novel allosteric small molecule inhibitors of CK2, azonaphthalene derivatives, has similar structures and ATP non-competitive features as hematein (27). The effect that these inhibitors have on CK2 is due to large conformational change of CK2α upon binding of these inhibitors. As a result, hematein may exert its inhibitory effect on CK2 through similar mechanisms. However, X-ray crystallographic analysis of the co-structure of CK2α-hematein complex will be required to precisely reveal the binding site of hematein.

In conclusion, we showed antitumor effects of hematein in A427 lung cancer cells and a xenograft nude mouse model of lung cancer. The therapeutic potential of hematein is emphasized by its efficacy at inhibiting lung cancer cells growth and inducing apoptosis. Furthermore, docking studies showed that hematein has durable binding sites to CK2 and may act as an allosteric inhibitor to CK2.
